# Ultrasound-assisted extraction of hemicellulose and phenolic compounds from bamboo bast fiber powder

**DOI:** 10.1371/journal.pone.0197537

**Published:** 2018-06-01

**Authors:** Cheng Wang, Claudia Tallian, Jing Su, Robert Vielnascher, Carla Silva, Artur Cavaco-Paulo, Georg M. Guebitz, Jiajia Fu

**Affiliations:** 1 International Joint Research Laboratory for Textile and Fiber Bioprocesses, Jiangnan University, Wuxi, China; 2 Institute for Environmental Biotechnology, Department for Agrobiotechnology (IFA-Tulln), University of Natural Resources and Life Sciences, Vienna (BOKU), Tulln an der Donau, Austria; 3 Centre of Biological Engineering, University of Minho, Campus de Gualtar, Braga, Portugal; 4 Austrian Centre of Industrial Biotechnology - ACIB, Tulln an der Donau, Austria; 5 Jiangsu Engineering Technology Research Center for Functional Textiles, Jiangnan University, Wuxi, China; College of Agricultural Sciences, UNITED STATES

## Abstract

Ultrasound-assisted extraction of hemicellulose and phenolic compounds from bamboo bast fibre powder was investigated. The effect of ultrasonic probe depth and power input parameters on the type and amount of products extracted was assessed. The results of input energy and radical formation correlated with the calculated values for the anti-nodal point (λ/4; 16.85 mm, maximum amplitude) of the ultrasonic wave in aqueous medium. Ultrasonic treatment at optimum probe depth of 15 mm improve 2.6-fold the extraction efficiencies of hemicellulose and phenolic lignin compounds from bamboo bast fibre powder. LC-Ms-Tof (liquid chromatography-mass spectrometry-time of flight) analysis indicated that ultrasound led to the extraction of coniferyl alcohol, sinapyl alcohol, vanillic acid, cellobiose, in contrast to boiling water extraction only. At optimized conditions, ultrasound caused the formation of radicals confirmed by the presence of (+)-pinoresinol which resulted from the radical coupling of coniferyl alcohol. Ultrasounds revealed to be an efficient methodology for the extraction of hemicellulosic and phenolic compounds from woody bamboo without the addition of harmful solvents.

## 1. Introduction

Bamboo is defined as a fast-growing lignocellulosic biomass, which is world widely distributed [1]. In general, plant biomass is mostly lignocellulosic and consists on cellulose, hemicellulose and lignin, which form the complex and rigid plant structure [2]. The formed complex matrix of the aromatic heteropolymer and hemicellulose account for about 18–40% of the dry weight of the plant. The structural variation of bamboo lignin of *Dendrocalamus brandisii*, which belongs to *Bambusoideae* of *Gramineae* and is mainly distributed in southeast Asia including the southwest region of China, has been studied by Bai et al. [3]. The main substructers of lignin in *D*. *brandisii* were identified by 2D HSQC NMR as being β-*O*-4′ aryl ether, resinol, spirodienone substructures, *p*-coumarate units, *p*-hydroxyphenyl units; guaiacyl units and syringyl units [3]. In addition to lignin, hemicellulose is a major component of wooden material, whereby galactoglucomannan is mainly present in native softwood and glucuronoxylans constitutes the main hemicellulose in native hardwood [4]. Recovery of both polymers, lignin and hemicellulose, by different extraction approaches gains increasing importance due to the various applications of these biomaterials. Hemicelluloses are used as raw materials of hydrogels for drug delivery or as oxygen barrier materials, while lignin derived compounds, like vanillin, *p*-coumaric acid and phenolic aldehydes, can be used as laccase mediators or as polymers on leather tanning [5][6].

The nature of molecules resulting from extraction of woody materials depends on the solvent used, amongst other parameters. Hot water extraction leads mainly to the release of hemicellulose and low molecular weight lignin. E. Maekawa studied the isolation and fractionation of water-soluble polysaccharides from bamboo shoots and was able to extract xylan, an arabinogalactan, and an α-glucan besides starch, using DMSO as solvent [7]. Song et al. used hot water at 170 °C for 60 min for the extraction of hemicelluloses from spruce wood [8]. Other methods for hemicellulose extraction from wood involving the use of harsh conditions, like steam explosion, treatment with alkali or dilute acid and hot-water, have been studied [8]. The extraction of lignin fractions from wood using solvents like ethanol has also been assessed. Phenolic compounds and aldehydes including vanillin, coniferyl aldehyde, syringaldehyde or *p*-hydroxycinnamylaldehyde were obtained by ethanolysis [9].

The potential of ultrasounds to assist the extraction of woody materials is largely unexplored, especially concerning bamboo. Ultrasound waves, through cavitation effects, can break vegetal material by directly disturbing the cell wall permeability and thereby enhance materials extraction [10]. It is expected that such ultrasound effects occur at low frequencies and high power. M. Vinatoru demonstrated the effect of frequency on the extraction efficiency of marigold leaves by the application of two different frequencies (20 and 500 kHz), whereby low frequency was found to destroy all excretion hairs (external oil glands) and part of the leaves [11]. The ultrasound effects are also related to the propagation of ultrasonic waves in the liquid medium producing local turbulences and micro-circulations, which is defined as acoustic streaming. This phenomena can enhance physical effects influencing the chemical processing, which is limited by mass transfer, e.g. reducing of chemical synthesis reaction time, increasing the effectiveness of the catalyst or switching the reaction pathways, resulting in increased selectivity[12][13]. Furthermore, the generation of free radicals, due to the dissociation of vapors trapped in the cavitating bubbles, may also occur. This results either in intensification of chemical reactions or in the propagation of certain reactions under ambient conditions[12]. The production of hydroxyl radicals during ultrasound cavitation might induce solubility of certain compounds of bamboo, which would be otherwise insoluble and hardly extractable in aqueous media.

In this study we aim to study the optimum reaction conditions for the ultrasound-assisted extraction of hemicellulose and lignin from bamboo in aqueous media. Parameters like ultrasonic path-length, frequency, power and temperature were optimized to achieve the highest levels of radical formation, which were characterized by dosimetric and calorimetric methods [14]. The effect of boiling as a bamboo pre-treatment on the final ultrasound-assisted extraction efficiencies was also evaluated The chemical composition of the extracts was analysed by LC-Ms-Tof.

## 2. Material and methods

### 2.1 Material

Commercial food grade bamboo poles obtained from Mount Huangshan, Anhui Province of China, were used in this study. 2-Hydroxyterephthalic acid (HTA), terephthalic acid (TA), conyferyl alcohol, sinapyl alcohol, D-xylobiose, vanillic acid, vanillin, gallic acid, 5-hydroxymethyl-furfural, D-cellobiose, D-cellotriose, glucuronic acid and guaiacol were purchased from Sigma Aldrich (USA). All other reagents were of analytical grade and used as received, if not otherwise specified.

### 2.2 Bamboo powder production

Bamboo poles were hand cut to chips for further treatment. Bamboo chips were grinded to powder using YF-1000 (Yongli, China) medicine pulveriser. The obtained powder was dried in an oven at 40 °C to constant weight and stored until further use.

### 2.3 Calorimetric characterization of ultrasound probe

For calorimetric characterization of the ultrasonic system the influence of the probe depth and the ultrasonic power, in distilled water, was evaluated at room temperature. Therefore, 5 mL of distilled water were sonicated at different power levels (200 and 400 W) using a ultrasonic probe with 3 mm diameter connected to a XO-SM50 sonicator with a frequency of 22 kHz (Nanjing Xian’ou Instrument Manufacturing Co. Ltd, China), testing different probe depths (5, 10, 15, 20 mm). Ultrasound was applied for a total time of 20 min. with a pulse ON time of 2 sec followed by a pulse OFF time of 4 sec. During the sonication process, temperature was measured using a inbuilt thermosensor. The input Energy (kJ) was calculated according to Eq 1.

$$E=\frac{\Delta T*m*C_{p}}{1000}$$ (1)

where *E* is the calculated input energy (kJ) to raise the water temperature; *ΔT* is the difference between the final and the initial temperature (K), *m* is the mass of the water (kg) and *Cp* is the heat capacity of water (4186 J kg^-1^ K^-1^) [15].

### 2.4 Dosimetric characterization

**Fig 1:**
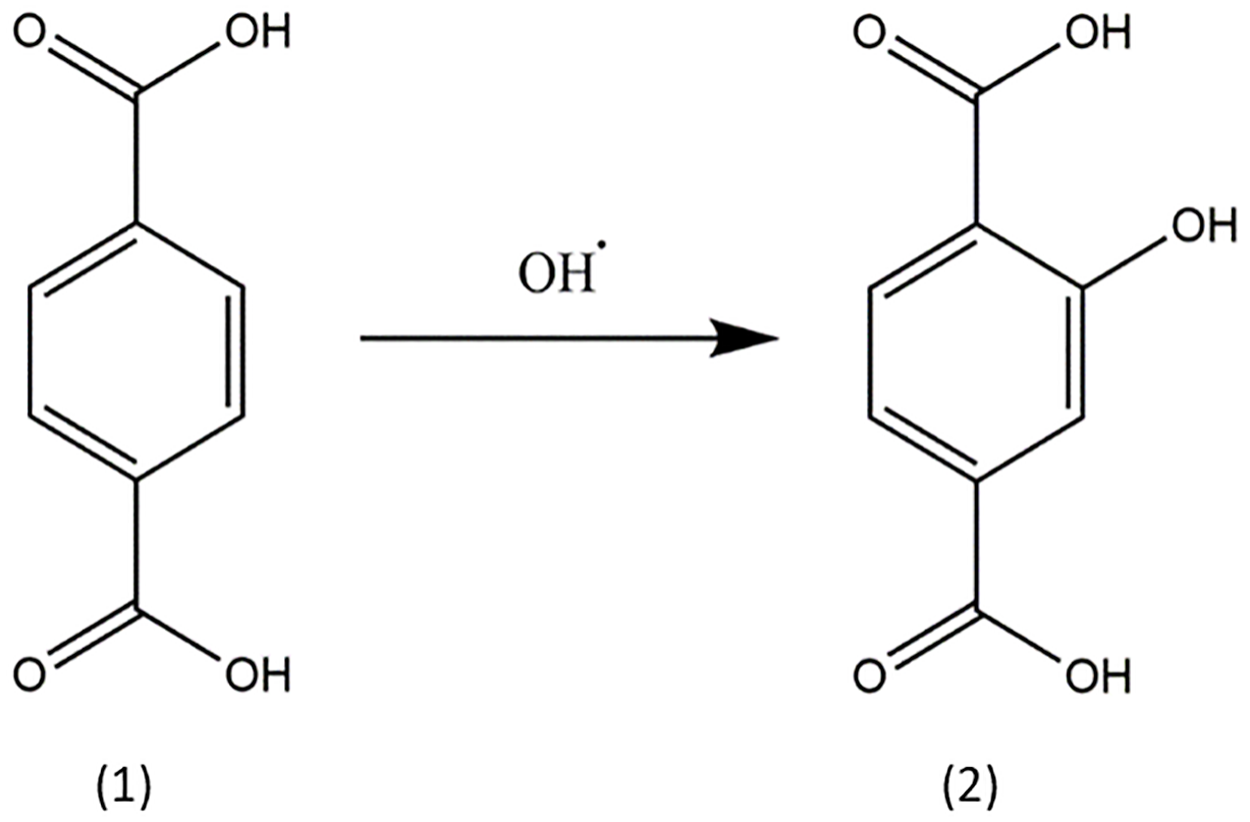
Radical conversion of (1) terephthalic acid to (2) 2-hydroxyterephthalic acid [16].

Dosimetric characterization of the ultrasonic-microwave reaction system was conducted by performing ultrasonic experiments at 22 °C. Therefore, the quantification of the produced hydroxyl radicals by acoustic cavitation was performed by conversion of the terephthalic acid (TA) into 2-hydroxyterephthalic acid (HTA) (Fig 1).

TA (0.3 mM) was dissolved in 0.1 M sodium phosphate buffer solution (pH 7.4) and 5 mL of the solution were subjected to sonication for 20 min. varying the probe depths (5, 10, 15, 20 mm) and the power intensities (200 and 400 W), respectively. Samples were subjected to ultrasound for 20 min. using a pulse ON time of 2 sec and a pulse OFF time of 4 sec. Afterwards, fluorescence spectroscopy was performed at an emission wavelength of 425 nm (λ_ex_ 315 nm) using EnSpire 2300 (PerkinElmer Instruments, Inc., China) fluorescence microplate reader. A calibration curve with different concentrations of 2-hydroxyterephthalic acid (0.2*10^-5^ mM, 2*10^-4^ mM, 4*10^-4^ mM, 6*10^-4^ mM, 8*10^-4^ mM, 10*10^-4^ mM) in 0.1 M sodium phosphate buffer (pH 7.4) was performed.

### Calculation of the ultrasound speed and the wavelength

The velocity (*c*) of ultrasound in pure water at 22 °C at a given input energy [kJ] was calculated from the Marczak equation (Eq 2) [16]. Based on the sound speed the wavelength was calculated using Eq 3.

$$c=1.402385\;\times 10^{3}\times \;t^{0}+5.038813\times 10^{0}\times t^{1}-5.799136\times 10^{-2}\times t^{2}+3.287156\times 10^{-4}\times t^{3}-1.398845\times 10^{-6}\times t^{4}+2.787860\times 10^{-9}\times t^{5}$$ (2)

$$\lambda =\frac{c}{f}$$ (3)

where *f* is the frequency (kHz) of the instrument.

### 2.5 Ultrasound-assisted extraction of hemicellulose and phenolic compounds from bamboo powder

To evaluate the influence of ultrasound on the extraction efficiency (EE%) of phenolic compounds from bamboo powder, experiments were performed at 40 °C using a XO-SM50 (Nanjing Xian’ou Instrument Manufacturing Co. Ltd, China) sonicator connected to a LTC2 combined refrigerated and heating bath circulator (Grant Instruments Ltd, UK). The materials extraction was firstly carried out on samples without pretreatment (named **Extraction A**, no pretreatment step). For each experiment, 0.8 g of bamboo powder was suspended in distilled water at final bath ratio of 1:10. Samples were incubated using a 15 mm ultrasonic probe depth and different input power levels (200 and 400 W). The total treatment time was set to 10 min using 2 sec pulse ON followed by 4 sec pulse OFF. For each sample five replicates were performed. As negative control, the extraction was performed without ultrasound. After treatments, all samples were centrifuged and the supernatant was separated from the solid and freeze-dried. The extraction efficiency (EE) was calculated by means of weighting of freeze-dried samples.

The effect of samples treatment prior ultrasound-assisted extraction was also evaluated. For this, 50 g of bamboo powder were suspended in 400 mL of distilled water and boiled for 60 min. The samples were centrifuged after each extraction step and the supernatants were collected for HPLC analysis. The remaining liquid was again freeze-dried and weight. The solid fraction was dried at 50 °C and used to conduct ultrasonic extraction (named **Extraction B**, one pretreatment step, followed by ultrasound extraction), by using 15 mm probe depth and input energies of 200 and 400 W. The remaining solid was used to performe another pretreatment followed by an extraction step (named **Extraction C**, two pretreatment steps followed by ultrasound extraction).

### 2.6 LC-ESI-TOF—Chemical analyses of the extracts

The chemical composition of the extracts produced was determined by LC-ESI-TOF (liquid chromatography coupled with electro spray ionization and time of flight mass spectrometry detection) measurements, using a 1260 HPLC (Agilent Technologies, US) equipped with a Poroshell 120 EC-C18 4.6 mm × 50 mm 2.7 Micron (Agilent Technologies, US) column. The injection volume was 30 μL. The diode array detector (DAD) was monitored at 254 nm linked to a Dual ESI TOF G6230B (Agilent Technologies, US). Electrospray ionization was operated in positive ion mode using a nebulizer with 8 L min^-1^ gas flow at 40 psig and 325 °C. Fragmentor voltage was set to 200 V, the skimmer at 100 V and the octupole to a voltage of 750 V. The reference masses were 121.0509 mz^-1^ and 922.0098 mz^-1^. Data were acquired in the mass range from 50–1000 mz^-1^ with the MassHunter workstation B08.00 (Agilent Technologies, US). For quantification standard substances for phenolic and hemicellulose components were measured under same conditions with a concentration range between 1 and 0.0001 mg/mL (see supplementary information, S2 Table).

## 3. Results and discussion

### 3.1 Calorimetric and dosimetric characterization of the ultrasonic reactor

**Fig 2:**
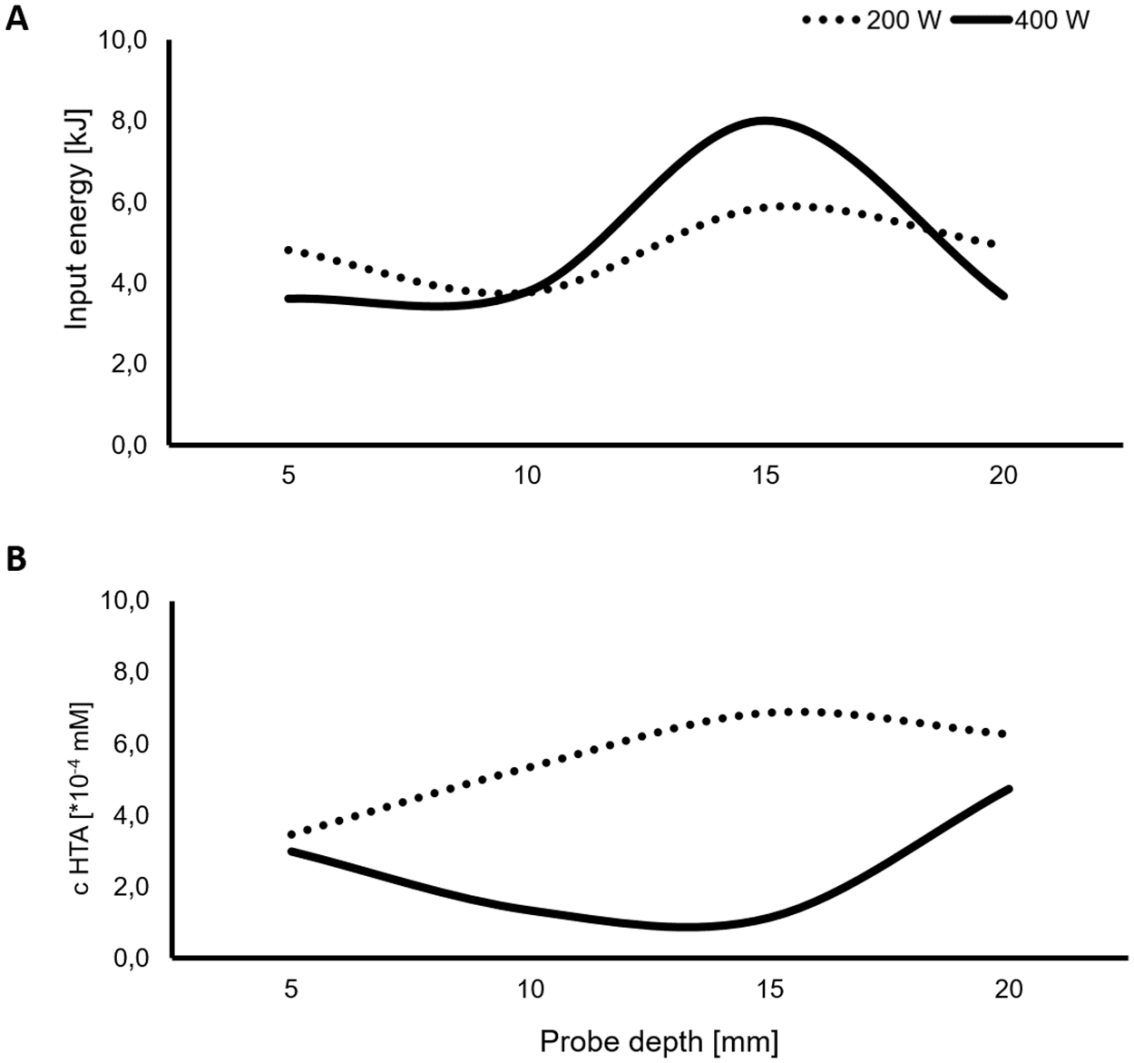
Variation of input energy [kJ] and HTA concentrations [*10^4^ mM] at different power inputs [W] of 200 and 400 W and probe depths [mm] of 5–20 mm.

Prior to ultrasound extraction of materials from bamboo powder, the reactor was characterized via calorimetric and dosimetric analysis testing different reactor probe depths. The sound wavelength was calculated to be 67.38 mm and consequently the anti-nodal point (maximum amplitude, λ/4) resulted at 16.85 mm and the nodal point (minimal amplitude, λ/2) at 33.69 mm. The input energy for the different power inputs tested (200 and 400 W) increased with the probe depth until reaching the theoretical anti-nodal point of the maximum amplitude of the wave (Fig 2A). The input energy for the calculated anti-nodal point 16.85 mm was not evaluated and the extraction was performed at the closed tested probe depth of 15 mm using the highest energy input (of 8 kJ), with a power input of 400 W.

The reactor was characterized via dosimetric method to evaluate the best conditions to produce the highest level of hydroxyl radicals [17]. Maximum hydroxyl radical formation was measured with a power input of 200 W and 15 mm probe depth (Fig 2B). Based on the calculated nodal and anti-nodal points of the wave, a maximum was expected near 16.85 mm probe depth and 400 W input energy comparable to the maximum input energy. This deviation between 200 and 400 W can be caused by different factors that may contribute to the heat energy of the solution leading to a decrease of the •OH radical production. Cavitation bubble implosions have a significant role regarding the hydroxyl radical formation. Heat energy contributors include cavitation bubble implosions of insufficient energy to form radicals, fluid friction within the solution due to mixing effects and friction between the solution and the stationary boundary layer adjacent to the vessel. These factors contribute in the rise of the heat energy even if the differentials in acoustic pressure are not providing effective caviation[18].

Further the optimal parameters which contribute for the highest radical formation and energy input were considered for the extraction of hemicellulose and phenolic lignin compounds from bamboo bast fibre. Ultrasonic-assisted treatment is expected to enhance diffusivity, mass transfer rates and the weaking of the cell walls leading to improved extraction[19][20].

### 3.2 Extraction efficiency and chemical analysis of extracted compounds

**Fig 3:**
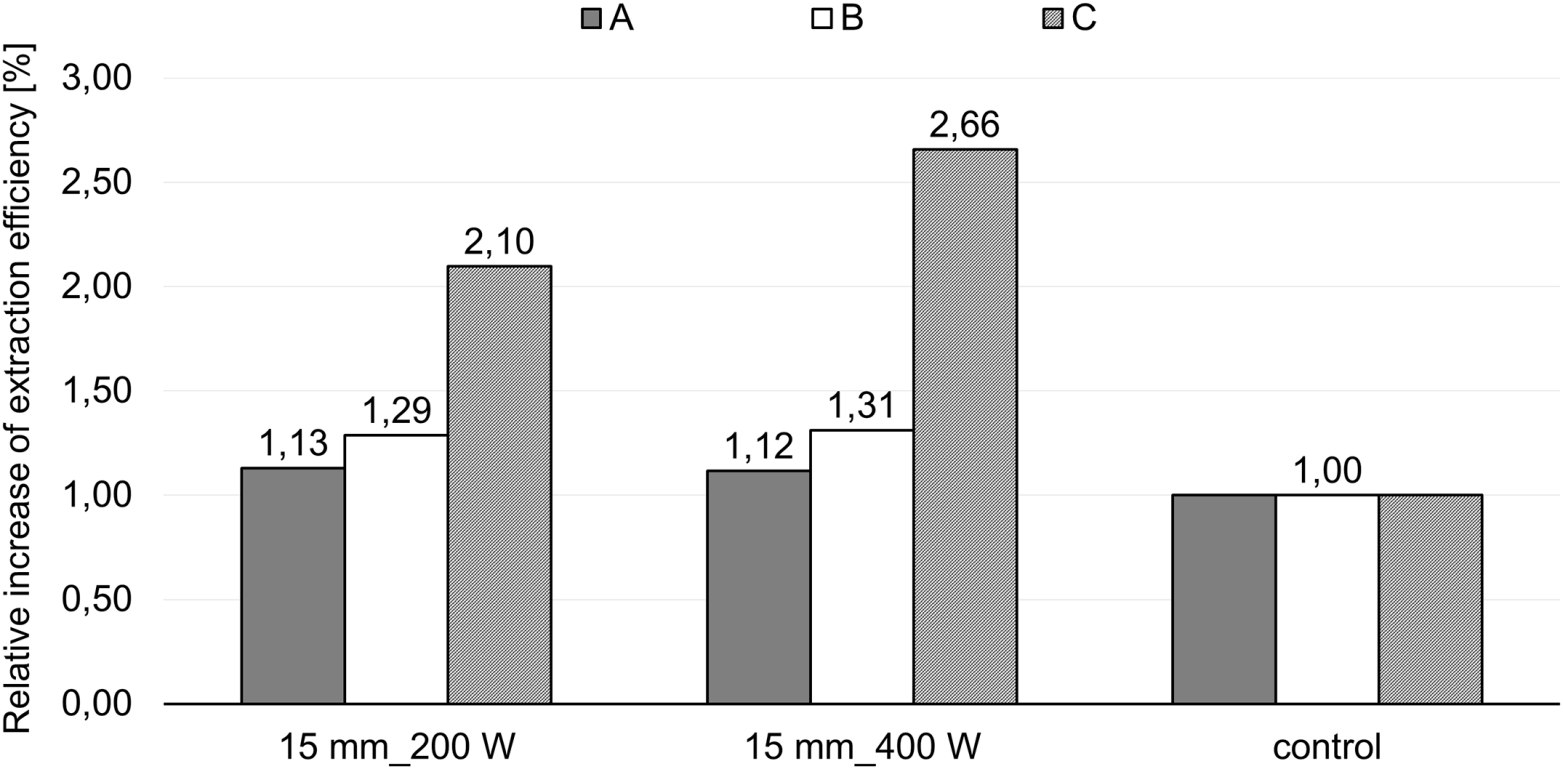
Normalized extraction efficiencies (%) for ultrasound-assisted extractions: (A) extraction without pretreatment, (B) extraction with one boiling water pretreatment and (C) extraction with two following boiling water pretreatments.

Based on the previous reactor characterisation, the extraction was carried out using the optimized conditions: 200W and 400W of input energy with a 15 mm probe depth and frequency of 22kHz. The efficiencies obtained for the different extraction conditions are presented in Fig 3 (see also S1 Table). The results reveal that ultrasound enhances the materials extraction in comparison with the control (water extraction only). The combination of ultrasonic extraction and boiling water pretreatment lead to a 2.6-fold increase of the extraction efficiency. This synergistic effect is more pronounced when extraction is performed after two boiling pretreatments (C) at 400W of input energy. One boiling pretreatment followed by ultrasound extraction gave riseto similar extraction efficiencies for both power inputs (A and B).

**Fig 4:**
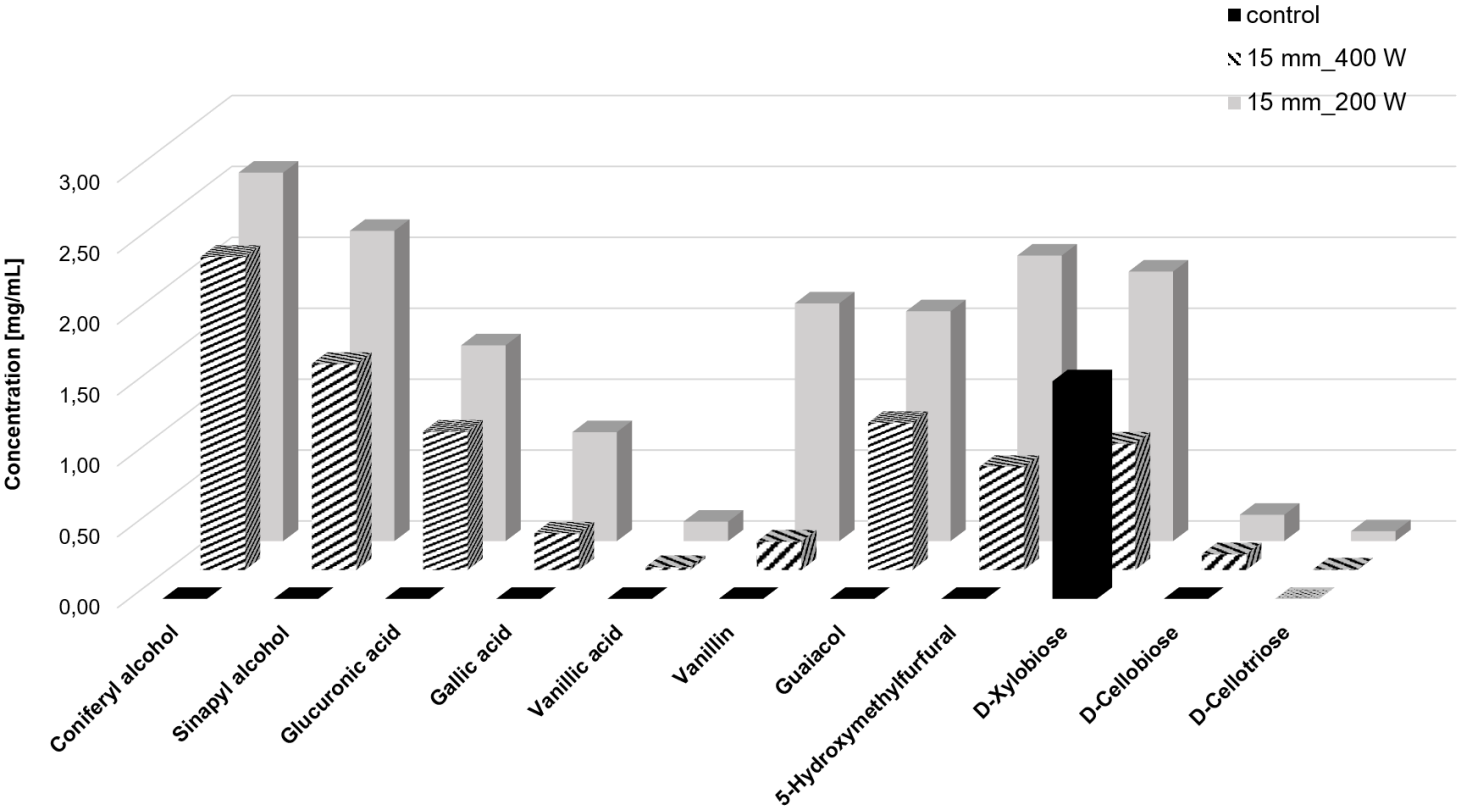
Summarised concentrations [mg/mL] of products extracted in two cycles from bamboo bast fibre powder at 200 and 400 W input power, with 15 mm probe depth, 22kHz, obtained by LC-ESI-TOF analyses.

After extraction procedures, the materials extracted were analysed and characterized by LC-ESI-TOF spectrometry (Fig 4).

According to LC-ESI-TOF analysis ultrasound-assisted extraction led to higher amounts of hemicellulose, phenolic components and molecules of lignin biosynthesis, when compared with hot water extraction only (Fig 4). Lignin monomers (LM) such as coniferyl alcohol and sinapyl alcohol, which are soluble in water were released in concentrations of up to 2.29 mg/mL in the second extraction step. At 200W higher concentration of products were extracted which can be correlated with the higher amount of radicals measured at this input energy. Vanillic acid, gallic acid, vanillin, guaiacol and 5-hydroxymethylfurfural, molecules involved in bamboo lignin biosynthesis [21], were also extracted in the presence of ultrasound. Independently on the extraction cycle, none of these phenolics was found after exteraction with water only (no ultrasound, see supplementary information, S2 Table). Ultrasound treatment increased cell wall permeability by the cavitation effects [11], namely cavitation bubble collapses near the cell walls disrupting the cell walls and enhancing their penetration without the addition of any solvent[10]. Cell wall disruption facilitates therefore the release of the extractable compounds enhencing mass transport [10]. This effect has been described by several authors, namely by Li et al. (2004) [22] for the extractions of oils from soybeans, by Ando et al. (2000) [23], Pingret et al. (2012) [24], Zhang et al. (2008) [25], for the ultrasound-assisted extraction and hot-compressed water extraction of plant materials.

In addition to phenolics, glucuronic acid, D-xylobiose and D-cellobiose, mono- and oligomers of bamboo hemicellulose components [26] were also successfully extracted. Bamboo lignin is described as classical grass lignin, built from *p*-coumaryl, coniferyl and sinapyl alcohol, which are interconnected. Due to the fast growth of Bamboo, lignification varies between the internodes[27]. The state of the art about the extraction of lignin from wooden material generally describes the use of acids in solvents[28], which are environmentally harmfull. In contrast to ultrasound extraction with water at neutral pH, hydrolysis based extraction methods usually require neutralization steps due to the acids and alkalis used. Moreover, corrosion effects are often disadvantages of these techniques [23], as well as the high amount of aggressive chemicals employed and discharged. The presented data indicates that ultrasound, enables the green extraction of LM in aqueous media without the addition of acidic solvents. Comparing with data reported, even using only water as solvent, similar levels of lignin and hemicelluloses extraction were achieved [29][30]. The ultrasound-assisted extraction method is seen therefore as promising alternative to conventional extraction procedures of plant materials.

**Fig 5:**
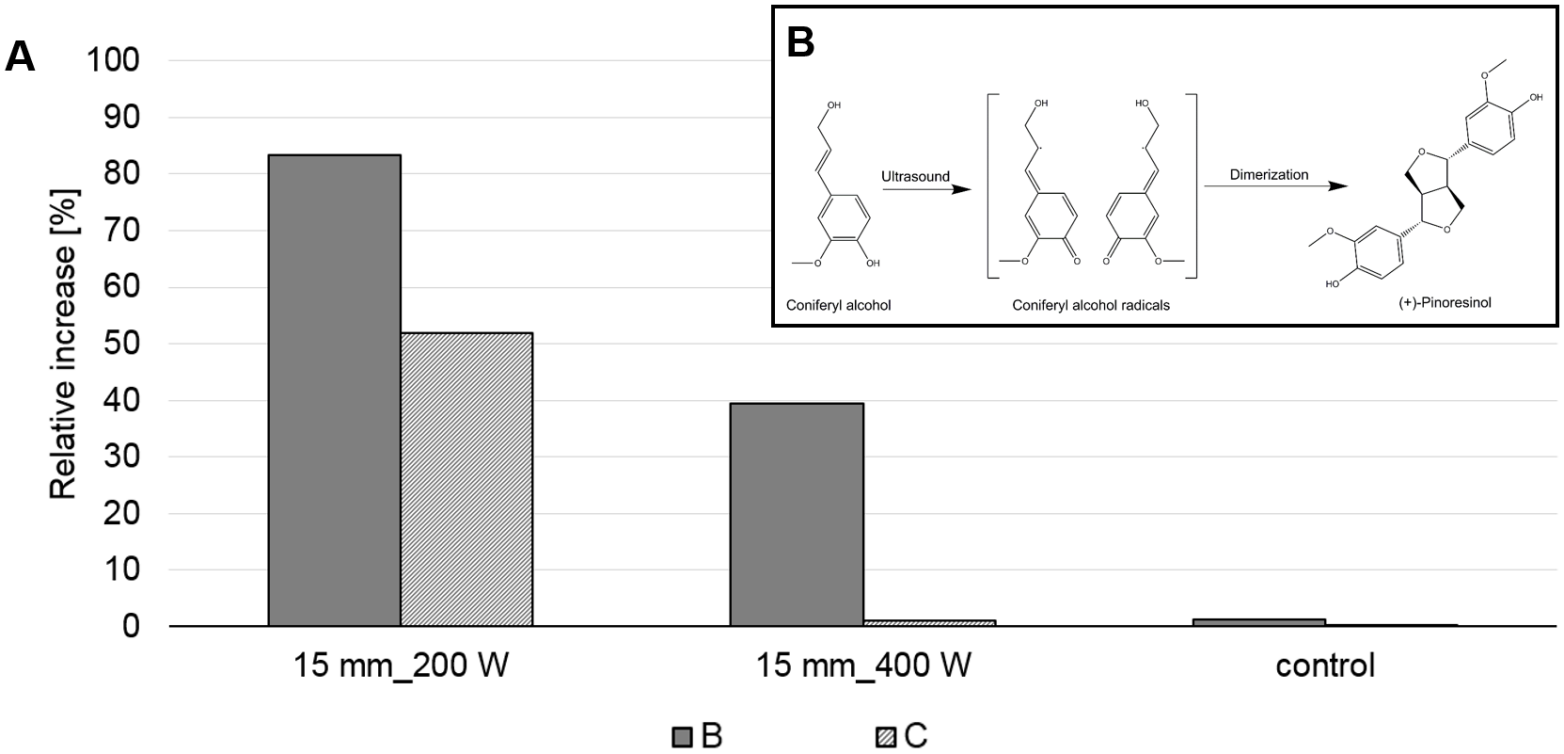
(A) Relative increase [%] of (+)-pinoresinol measured by LC-ESI-TOF after extraction B (extraction with one boiling water pretreatment) and C (extraction with two following boiling water pretreatments) using an input power of 200 or 400 W with a 15 mm probe depth (B) Dimerization reaction of coniferyl alcohol radicals generated by ultrasound induced radical formation to (+)-pinoresinol; the control consisted on the extraction without ultrasound.

Additionally to an efficient extraction, the dimerization of coniferyl alcohol to form a lignal, (+)-pinoresinol, was investigated [31][32] (Fig 5). The highest amount of (+)-pinoresinol (83%) was detected after the second extraction step at the optimum parameters for radical formation (15 mm probe depth and 200 W input power) (Fig 5A). The effective radical (+)-pinoresinol dimerization confirms the optimum conditions for radical formation determined previously by dosimetric method. The possibility of radical polymerization exploited herein can be further exploited using laccase as catalyst after extraction. This promising ability open-up the way for a vast amount of products that can be extracted and polymerized using green treatment conditions.

## 4. Conclusion

Probe depth and input energy are key parameters to tune the ultrasound- assisted extraction of hemicellulose and lignin based compounds from bamboo bast fibre powder. The optimum settings calculated from models were verified based on the quantification of radicals produced, while additional effects such as heat energy seemed also to play a role in the extraction process. Extraction efficencies increased by 2.6-fold for ultrasound treated samples comparing with extraction only with water (control). The chemical analysis of the extracted fractions by LC-ESI-TOF indicated that the application of ultrasound led to the release of higher amount of lignin monomers, molecules of the lignin biosynthesis and hemicellulotic compounds. The effect of ultrasound is directly correlated with the radical generation, as well as with the increase of the cell wall permeability and mass transfer phenomena promoted by the cavitation effects near the cell walls. Our findings reveal ultrasound as a green technology for the extraction of bamboo components without solvent addition.

## Supporting information

S1 Table Original data of extraction efficiencies [%] calculated as weight fractions of the freeze dried liquids after the extractions A, B and C. (DOCX)

S2 Table Standard substances tested by LC-ESI-TOF for quantitative analysis. (DOCX)
