# Supplementary material for: Ultrasound-assisted extraction of hemicellulose and phenolic compounds from bamboo bast fiber powder
Source: PLoS One. 2018 Jun 1;13(6):e0197537. doi: 10.1371/journal.pone.0197537 (PMC5983477; doi:10.1371/journal.pone.0197537)
Supplement: S1 Table — (DOCX) [file pone.0197537.s001.docx]

**Supporting information**

**S1 Table:** Original data of extraction efficiencies [%] calculated as weight fractions of the freeze dried liquids after the extractions A, B and C

|  | Extraction A  EE [%] | Extraction B  EE [%] | Extraction C  EE [%] |
| --- | --- | --- | --- |
| 15 mm_200 W | 2.6750±0.50 | 1.1550±0.49 | 0.5375±0.13 |
| 15 mm_400 W | 2.6400±0.48 | 1.1775±0.49 | 0.6813±0.13 |
| control | 2.3650±0.47 | 0.8975±0.37 | 0.2563±0.11 |
