# Supplementary material for: Ultrasound-assisted extraction of hemicellulose and phenolic compounds from bamboo bast fiber powder
Source: PLoS One. 2018 Jun 1;13(6):e0197537. doi: 10.1371/journal.pone.0197537 (PMC5983477; doi:10.1371/journal.pone.0197537)
Supplement: S2 Table — (DOCX) [file pone.0197537.s002.docx]

**S2 Table:** Standard substances tested by LC-ESI-TOF for quantitative analysis

| Nr. | Phenolics  (P) | Lignin Monomers  (LM) | Mono-saccharides  (MS) | Di-saccharides  (DS) | Oligo-saccharides  (OS) | Acids  (AC) | Alcohols  (AL) |
| --- | --- | --- | --- | --- | --- | --- | --- |
| I | 5-(hydroxymethyl)furan-2-carbaldehyde | 4-(3-hydroxy-1-propenyl)-2-methoxyphenol (coniferyl alcohol) | D-Glucose | D-Xylobiose | D-Cellotriose | 3,4,5-trihydroxybenzoic acid  (gallic acid) | benzene-1,2-diol  (catechol) |
| II |  | 4-(3-hydroxyprop-1-enyl)-2,6-dimethoxyphenol (sinapyl alcohol) | L-Arabinose | D-Cellobiose |  | 4-hydroxy-3-methoxybenzoic acid (vanillic acid) | 2-methoxyphenol (guaiacol) |
| III |  |  | D-Xylose | D-Saccharose |  | (2*S*,3*S*,4*S*,5*R*,6*R*)-3,4,5,6-tetrahyroxyoxane-2-carboxylic acid (glucuronic acid) |  |
| IV |  |  | D-Galactose |  |  |  |  |
| V |  |  | D-Mannose |  |  |  |  |
